# Re‐emergence of influenza virus circulation during 2020 in parts of tropical Asia: Implications for other countries

**DOI:** 10.1111/irv.12844

**Published:** 2021-02-10

**Authors:** Joshua A. Mott, Alicia M. Fry, Rebecca Kondor, David E. Wentworth, Sonja J. Olsen

**Affiliations:** ^1^ Influenza Division Centers for Disease Control and Prevention Regional Influenza Program Bangkok Thailand; ^2^ Influenza Division Centers for Disease Control and Prevention Atlanta GA USA

**Keywords:** H3N2 subtype, Influenza A virus, sentinel surveillance, Southeastern Asia, tropical climate

Global influenza virus circulation declined and has been below traditional seasonal levels during the COVID‐19 pandemic.^1^, ^2^, ^3^ After learning of increased influenza virus circulation in some tropical Asian countries, we reviewed current surveillance data to better ascertain risk for an influenza resurgence during the 2020‐2021 Northern Hemisphere influenza season and subsequent seasons during the COVID‐19 pandemic.

We reviewed WHO influenza surveillance outputs from May 1 to December 31, 2020 (epidemiologic weeks 18‐53) from tropical Asian countries that have land mass between the Tropics of Cancer and Capricorn.^4^ We excluded China and Australia, which have influenza seasons that generally follow temperate seasonal patterns. We identified countries that tested surveillance specimens for influenza viruses ≥50% of the 36 weeks (19/36 weeks). For each country, we report influenza surveillance specimens tested, and the percentage positive for influenza, by type and subtype; we compared current data to historical data from 2015 to 2019.

Of 17 countries, 12 (70%) reported testing influenza surveillance specimens for more than half of the weeks during May 1 to December 31, 2020 (Table 1). These 12 countries tested 17,407 surveillance specimens, with 592 (3.4%) positive for influenza viruses. Influenza A(H3N2) viruses were detected in 573/592 (97%) influenza positive specimens. From April 27 to July 26, 2020 (epidemiologic weeks 18‐30), specimens tested decreased from an average of 14,102 per year in 2015‐2019 to 3,969 (71.9% decrease) and influenza positivity from 22% to ≤ 1%. During weeks 31‐53, specimens tested decreased from an average of 24,782 per year in 2015‐2019 to 13,438 (45.8% decrease) and influenza positivity from 18% to 4%.

**TABLE 1:** Laboratory‐confirmed influenza reported to WHO/FluNet among tropical Asian countries that maintained influenza surveillance during 2020: Epidemiologic weeks 18‐53, 2015‐2020

Country	Influenza virus circulation, 2020								Influenza virus circulation, 2015‐2019	
	Most recent week of report	Most recent week of influenza detection	Number of weeks with specimens processed (% of 36 weeks)	Specimens tested during epidemiologic weeks 18‐53	Influenza detected during weeks 18‐53^a^	Epidemiologic weeks during weeks 18‐53, with influenza virus detections	Influenza surveillance specimens tested and number positive in weeks 18‐30	Influenza surveillance specimens tested and number positive in weeks 31‐53	Influenza surveillance specimens tested and number positive in weeks 18‐30	Influenza surveillance specimens tested and number positive in weeks 31‐53
Bangladesh	52	45	35 (97%)	3,552	208 A(H3)	25, 32‐45	1/745 (0.1%)	207/2,807 (12%)	3,420/10,591 (32%)	1,151/13,954 (8%)
Cambodia	52	46	35 (97%)	737	108 A(H3)	27,29, 31‐46	3/285 (1%)	105/452 (23%)	463/1,663 (28%)	819/2,957 (28%)
India	51	51	30 (83%)	2,176	13 A(H3)	41‐45, 47, 50‐51	0/574 (0%)	13/1,602 (1%)	1,825/12,889 (14%)	5,013/33,936 (15%)
Indonesia	41	36	22 (61%)	131	3 A(H3)	20, 34, 36	1/66 (2%)	2/65 (3%)	769/3,784 (20%)	1,205/5,958 (20%)
Lao PDR	52	51	34 (94%)	2,205	116 A(H3) 1 A(H1)	32, 36, 38‐51	0/390 (0%)	117/1,815 (6%)	447/6,459 (7%)	2,103/10,405 (20%)
Malaysia	50	34	31 (86%)	1,607	1 A(H1)	34	0/563 (0%)	1/1,044 (0.1%)	653/5,790 (11%)	1,042/8,239 (13%)
Maldives	53	n/a	20 (56%)	138	0	n/a	0/47 (0%)	0/91 (0%)	419/1,334 (31%)	191/1,332 (14%)
New Caledonia	53	n/a	36 (100%)	462	0	n/a	0/236 (0%)	0/226 (0%)	489/2,098 (23%)	514/2,495 (21%)
Singapore	53	n/a	36 (100%)	1,497	0	n/a	0/398 (0%)	0/1,099 (0%)	1,951/4,217 (46%)	1,536/4,645 (33%)
Thailand	43	43	26 (72%)	741	3 A(H1) 1 B	36,37,43	0/252 (0%)	4/489 (1%)	1,033/3,939 (26%)	2,628/8,231 (32%)
Timor‐Leste	47	46	21 (58%)	2,772	14 A(H3) 12 A(H1) 1 B	30, 32‐33, 38‐42, 44, 46	8/49 (16%)	19/2,723 (1%)	99/293 (34%)	4/347 (1%)
Viet Nam	50	50	33 (92%)	1,389	111 A(H3	40‐50	0/364 (0%)	111/1,025 (11%)	848/3,349 (25%)	1,276/6,627 (19%)
Total				17,407	573 A(H3) 17 A(H1) 2 B		13/3,969 (0.3%)	579/13,438 (4%)	12,516/56,406 (22%)	17,482/99,126 (18%)

Note:

Brunei (not in WHO FluNet), Myanmar (11 weeks), Papua New Guinea (0 weeks), Philippines (0 weeks), and Sri Lanka (3 weeks) did not report over 50% of weeks with influenza specimens processed to meet inclusion criteria (data last accessed on January 15, 2021).

^a^ The years 2015 and 2020 included 53 epidemic weeks. The years 2016‐2019 included 52 epidemic weeks.

Six countries (Bangladesh, Kingdom of Cambodia, Lao PDR, New Caledonia, Singapore, and Viet Nam) maintained testing of surveillance specimens for >90% of weeks. In these countries, we observed considerable variability in influenza circulation. Influenza circulation was unseasonably low, or absent, during weeks 18‐30, 2020. However, during weeks 31‐53, the percentage of surveillance specimens testing positive for influenza approached or reached positivity rates of 2015‐2019 in Bangladesh and Cambodia and increased but remained lower than historical positivity in Lao PDR and Viet Nam. No influenza viruses were reported by New Caledonia and Singapore.

Many countries in tropical Asia are struggling to conduct influenza surveillance in 2020, likely due to disruptions from the COVID‐19 pandemic. However, unlike the observation in the Southern Hemisphere^1^, ^5^, ^6^ influenza has not disappeared from circulation, and some countries experienced a delayed resurgence of community circulation of influenza viruses. The heterogeneity in the magnitude of the influenza activity across countries in tropical Asia is noteworthy and likely due to multiple factors, including surveillance artifact, degree of travel restrictions, and adherence to COVID‐19 interventions. This has important implications for the 2020‐2021 Northern Hemisphere influenza season, and for future influenza vaccine strain selection.

First, it is critical that persons recommended to receive influenza vaccinations get vaccinated. The COVID‐19 pandemic is disproportionately impacting older persons, those with underlying chronic conditions, and other traditionally underserved populations,^7^, ^8^ many of whom are also at increased risk of severe complications of influenza. Influenza vaccination could prevent unnecessary morbidity and mortality and ease the strain on healthcare facilities.

Second, maintaining surveillance and outbreak response is essential to track the geographic spread and obtain viruses so vaccines remain optimized to circulating influenza viruses. At the WHO Collaborating Center for Reference and Research on Influenza at CDC Atlanta, global surveillance submitted 583 influenza viruses to CDC between March 1 and September 30, 2020, a 69% decrease from an average of 1908 viruses during the same months in the years 2015‐2019. Furthermore, the limited data from tropical Asia suggest that genetically divergent influenza A(H3N2) viruses are currently in circulation. Some viruses from Cambodia and Bangladesh belong to a different genetic clade, 3C.2a1b + T131K, than the currently recommended 2020‐2021 Northern Hemisphere influenza vaccine virus, A/Hong Kong/267/2019, which comes from genetic clade 3C.2a1b + 135K+137F.^9^

The data presented here are a reminder that the low levels of influenza circulation seen in the Northern Hemisphere in summer 2020 may not necessarily persist into the upcoming influenza season, and influenza surveillance and prevention strategies should continue as planned and not be delayed.

## CONFLICT OF INTEREST

The findings and conclusions in this report are those of the authors and do not necessarily represent the official of the Centers for Disease Control and Prevention.

## AUTHOR CONTRIBUTION

**Joshua Mott:** Conceptualization (equal); Data curation (lead); Formal analysis (lead); Validation (lead); Writing‐original draft (lead); Writing‐review & editing (lead). **Alicia M. Fry:** Conceptualization (equal); Funding acquisition (equal); Supervision (lead); Writing‐review & editing (equal). **Rebecca JG Kondor:** Data curation (supporting); Formal analysis (supporting); Validation (equal); Writing‐original draft (supporting); Writing‐review & editing (equal). **David Wentworth:** Data curation (equal); Formal analysis (supporting); Supervision (equal); Writing‐original draft (supporting); Writing‐review & editing (supporting). **Sonja J. Olsen:** Conceptualization (equal); Data curation (equal); Formal analysis (equal); Methodology (equal); Supervision (equal); Validation (equal); Writing‐original draft (equal); Writing‐review & editing (equal).

## Data Availability

Data are publicly available on WHO FluNet already.
